# Supplementary figures and images for: Linking neural and clinical measures of glaucoma with diffusion magnetic resonance imaging (dMRI)
Source: PLoS One. 2019 May 31;14(5):e0217011. doi: 10.1371/journal.pone.0217011 (PMC6544345; doi:10.1371/journal.pone.0217011)

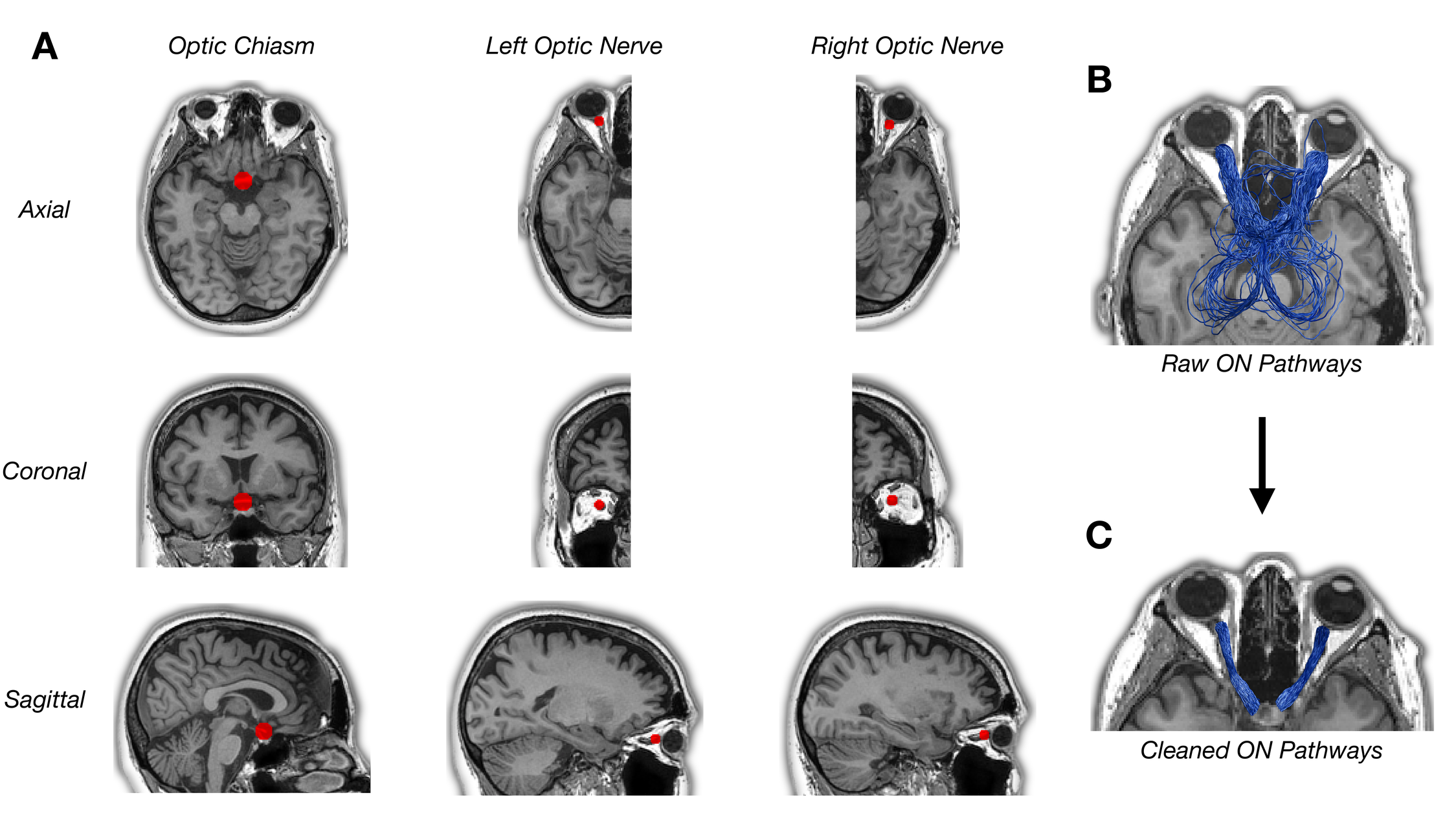

Supplement: S1 Fig — (A) Optic chiasm (6-mm sphere) and left and right optic nerve (4-mm spheres) regions of interest (ROI) placement in axial, coronal, and sagittal views (top to bottom). ROIs drawn in red. (B) Visualization of pre-cleaned tractography-generated left and right optic nerve white-matter pathways (blue). (C) Visualization of cleaned tractography-generated left and right optic nerve white-matter pathways (blue). (TIF) [file pone.0217011.s001.tif]

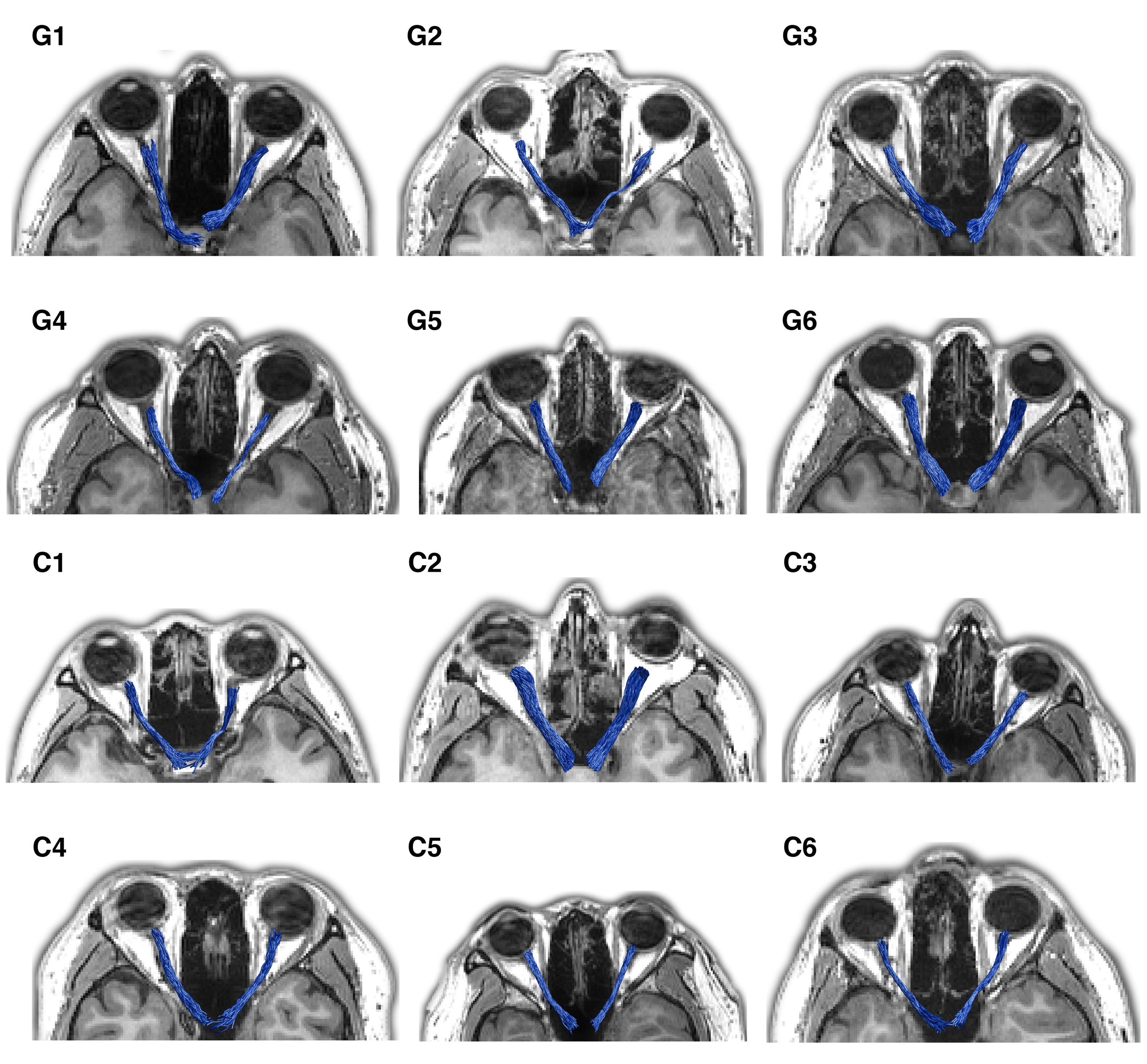

Supplement: S2 Fig — Visualization of final tractography-generated left and right optic nerve white-matter pathways (blue) in six glaucoma patients (denoted G1-G6) and six control subjects (denoted C1-C6). (TIF) [file pone.0217011.s002.tif]
