# Supplementary material for: Linking neural and clinical measures of glaucoma with diffusion magnetic resonance imaging (dMRI)
Source: PLoS One. 2019 May 31;14(5):e0217011. doi: 10.1371/journal.pone.0217011 (PMC6544345; doi:10.1371/journal.pone.0217011)
Supplement: S1 Table — Detailed description of the specific fiber cleaning results for six glaucoma patients (denoted G1-G6) and six control subjects (denoted C1-C6). The total number of fibers removed, percentage of fibers retained, and the normalized pathway volumes of the left and right optic nerves of all glaucoma patients and control subjects are provided. Values corresponding to the “advanced” glaucomatous eyes are marked in bold. Pathway volume estimates were generated using AFQ and are based on the number of unique fiber coordinates at each sample point. The number of fibers removed, percentage retained, and the normalized pathway volumes varied between subjects based on the number of initial fiber streams isolated. There was not a significant difference between average normalized volumes of glaucomatous and healthy control optic nerves (t(1,22) = -0.4185, p = 0.68). Optic nerve volumes across all subjects were found to be normally distributed in the Shapiro-Wilk test (p > 0.05) [42] and no significant outlier volumes were found with Iglewicz and Hoaglin’s outlier test (all modified Z scores < 3.5) [43]. (DOCX) [file pone.0217011.s003.docx]

|  | **Left ON** | **Left ON** | **Left ON** | **Right ON** | **Right ON** | **Right ON** |
| --- | --- | --- | --- | --- | --- | --- |
| **Subject** | ***# Fibers Removed*** | ***% Fibers Retained*** | ***Normalized Volume*** | ***# Fibers Removed*** | ***% Fibers Retained*** | ***Normalized Volume*** |
| G1 | **1718** | **4.50%** | **3609** | 20979 | 5.88% | 6550 |
| G2 | 7682 | 2.15% | 2705 | **508** | **3.24%** | **982** |
| G3 | 3594 | 4.01% | 4243 | **12326** | **5.74%** | **7238** |
| G4 | 10370 | 2.80% | 2641 | **1634** | **1.86%** | **1074** |
| G5 | **20954** | **5.28%** | **5732** | 117911 | 7.97% | 8820 |
| G6 | **24679** | **4.01%** | **5897** | 102278 | 7.92% | 7298 |
| C1 | 120005 | 0.44% | 6656 | 44070 | 0.07% | 1995 |
| C2 | 82708 | 6.79% | 10096 | 179784 | 8.22% | 11857 |
| C3 | 59191 | 0.22% | 4364 | 43552 | 0.45% | 4157 |
| C4 | 15782 | 0.32% | 2974 | 56215 | 0.18% | 5028 |
| C5 | 129137 | 0.13% | 4838 | 122713 | 0.09% | 3957 |
| C6 | 86704 | 0.10% | 3220 | 69974 | 0.17% | 3333 |
